# Acoustic Metasurface-Aided Broadband Noise Reduction in Automobile Induced by Tire-Pavement Interaction

**DOI:** 10.3390/ma14154262

**Published:** 2021-07-30

**Authors:** Hyeonu Heo, Mathew Sofield, Jaehyung Ju, Arup Neogi

**Affiliations:** 1 Department of Physics, University of North Texas, P.O. Box 311427, Denton, TX 76203, USA; hyeonu.heo@unt.edu (H.H.); mathewsofield@my.unt.edu (M.S.); 2 UM-SJTU Joint Institute, Shanghai Jiao Tong University, 800 Dongchuan Road, Shanghai 200240, China

**Keywords:** acoustic metasurfaces, tire cavity, noise reduction, acoustic metamaterials

## Abstract

The primary noise sources of the vehicle are the engine, exhaust, aeroacoustic noise, and tire–pavement interaction. Noise generated by the first three factors can be reduced by replacing the combustion engine with an electric motor and optimizing aerodynamic design. Currently, a dominant noise within automobiles occurs from the tire–pavement interaction over a speed of 70–80 km/h. Most noise suppression efforts aim to use sound absorbers and cavity resonators to narrow the bandwidth of acoustic frequencies using foams. We demonstrate a technique utilizing acoustic metasurfaces (AMSes) with high reflective characteristics using relatively lightweight materials for noise reduction without any change in mechanical strength or weight of the tire. A simple technique is demonstrated that utilizes acoustic metalayers with high reflective characteristics using relatively lightweight materials for noise reduction without any change in mechanical strength or weight of the tire. The proposed design can significantly reduce the noise arising from tire–pavement interaction over a broadband of acoustic frequencies under 1000 Hz and over a wide range of vehicle speeds using a negative effective dynamic mass density approach. The experiment demonstrated that the sound transmission loss of AMSes is 2–5 dB larger than the acoustic foam near the cavity mode, at 200–300 Hz. The proposed approach can be extended to the generalized area of acoustic and vibration isolation.

## 1. Introduction

Noise pollution by traffic is the most widespread environmental problem that causes sleep disturbance, hearing damage, and even cardiovascular disease [1,2]. Thus, the European Parliament and the Member States agreed to reduce road noise in 2011, introduced noise regulations, and reduced noise levels by just 3 dB (reducing sound pressure energy to half) in 2013 [3]. The primary noise sources of vehicles are engine, exhaust, aeroacoustic noise, and tire–pavement interactions [4]. Noise generated by the first three factors can be essentially reduced by replacing the combustion engine with an electric motor and optimizing aerodynamic design. However, reducing Tire–Pavement Interaction Noise (TPIN), which dominates the total noise generated within a vehicle at a speed of over 70–80 km/h [5,6], is hugely challenging. Any proposed technique should consider the structural and mechanical integrity of the tires and wheels, which can be problematic because the tire cavity environment is affected by changes in loading conditions, speed, and temperature [7]. Tire and automobile manufacturers are developing soundproofing techniques using resonators and sound-absorbers that meet the new industry standards [3] to provide a comfortable feeling for passengers.

Compressed air in a tire cavity generates resonant noise and vibration at a frequency range below 1,000 Hz, and the fundamental frequency is near 230 Hz [8]. Conventional noise reduction techniques using thick metal plates are not applicable for isolating this resonant noise and vibration of compressed air in a tire cavity due to design constraints. Tire manufacturers and researchers add a polyurethane absorber glued around the tire′s inner liner to reduce noise [9,10]. A resonator attached to the rim has been used to reduce the tire cavity′s resonant sound [11,12,13]. The pitch arrangement of the tire tread patterns has also been optimized to minimize the noise [14,15]. However, these noise reduction methods mainly focused on the resonator for the tire cavity resonance, i.e., a narrow bandwidth sound absorption capability of porous materials. A different approach is necessary for better noise reduction of the sound generated at low frequency and the wideband noise caused by tire–pavement interaction. 

Acoustic metasurfaces (AMSes) or metalayers are artificially designed 2D materials of subwavelength thickness that provide a non-trivial local phase shift and alter the direction of propagation of the incident wave. Some recent reports utilize AMSes for extraordinary sound absorption [16] using Helmholtz resonators [17], membranes [18,19,20], and 3D space coiling metastructures [21]. The membranes have shown over 200 times noise reduction for a specific frequency range and can be optimized for the desired application by modifying the geometry [18]. Lightweight yet soundproof acoustic metasurfaces were used within an airplane framework to reduce noise [20]. The structure consisted of a perforated, stiff, periodic pattern and thin, soft materials on a periodic structure. AMSes can be designed with a negative effective dynamic mass density $\left(\rho_{eff}<0\right)$ when the frequency is below the fundamental frequency of a thin plate. AMSes provide anti-resonance, out of phase with the incident wave and exponential decaying wave $\left(\Delta d\propto \left|\rho_{eff}^{-1/2}\right|\right)$, resulting in almost total reflection at the low broad frequency ranges [18,19]. The proposed AMSes may be used as an alternative method for noise reduction in tires.

In this study, the AMSes were designed to maximize the sound transmission loss (STL) over a broad frequency range using lightweight materials without modifying the mechanical properties of the tire. The noise reduction in the tire was investigated using AMSes based on hexagonal unit cells attached to the rim′s circumference to reflect sound waves arising from the tire–pavement interaction aided by the absorption in the radial direction. The AMSes were optimized for a particular design parameter with a negative effective dynamic mass density for a practical car tire. Based on the parametric study of the unit cell of AMSes, the noise reduction capability of AMSes is demonstrated through static tests using the tire cavity model and through a dynamic field test. The acoustic foam was considered as a comparison. The results showed that AMSes have 2–3 times (3 dB) more noise reduction capability than foam.

Within a few decades, various acoustic metamaterials have been proposed and their remarkable properties have been investigated extensively, e.g., complete bandgap at a particular band [22,23], or nonreciprocity (one-way transmission) [24,25]. However, the proven practical applications are spare and challenging due to the design constraints. Here, we first applied acoustic metasurfaces to reduce unwanted noise in the cabin. The proposed design, a lightweight and thin structure, does not affect the tire′s performance. Unlike other commonly used soundproofing methods, which have absorption capabilities of about 0.4~0.6 [26], AMSes show almost total reflection at low frequency, under 1000 Hz. Furthermore, AMSes can be easily combined with existing technologies, e.g., acoustic foam and optimal tread pitch patterns, to maximize the sound transmission loss in the cabin. The potential applications of the proposed technology are not limited to automobiles or tires. The robust and lightweight design can be modified and applied to other fields for soundproofing and vibration isolation to reduce undesirable noise issues.

## 2. Design and Fabrication of AMS

### 2.1. Design

The noise generated by tire–pavement interaction is the structure-borne noise at low-frequency ranges (below 500 Hz), while the air-borne noise occurs at high-frequency ranges (500–2000 Hz). According to Chang et al. [27], TPIN becomes the primary source of noise occurring at frequencies below 500 Hz, part of the audible frequency range. The fundamental frequency, *f*, of the tire cavity is a function of the speed of sound of air, *c*, and wavelength, *λ*; $f=c/\lambda =2c/\pi \left(D_{o}+D_{i}\right)$ where *D*_o_ is the outer diameter of the tire cavity toroid, and *D*_*i*_ is the inner diameter of tire cavity toroid [8]. For general passenger vehicles, the cavity mode is close to 230 Hz, which needs to be reduced [28,29].

**Figure 1:**
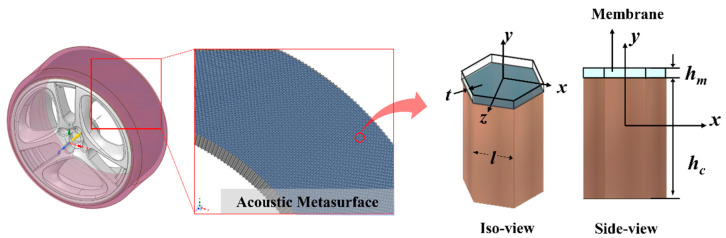
A concept to reduce tire noise with an acoustic metasurface consisting of a honeycomb core panel and a soft, thin plate on the tire′s rim.

Highly reflective AMSes were specifically designed for this frequency range, as shown in Figure 1. AMSes are fabricated using silicone rubber and are composed of a honeycomb-shaped core panel attached to a tire′s rim. In Figure 1, for the unit cell, *a_m_* is the side length of the hexagon, *t* is the wall thickness of the core panel, and *h_m_* and *h_c_* are the thickness of the thin plate and the height of the core panel, respectively. Hexagonal unit cell-shaped metasurfaces have a natural oscillation mode at higher frequencies than squares and triangles with identical unit cells with hydraulic diameters. Moreover, the shape and form of the periodic metasurfaces with hexagonal unit cells are not deformed across a tire’s curved plane. This also offers the best surface filling fraction, which is ideal for noise suppression. The unit cell can be considered as a clamped thin plate because the core panel is relatively rigid. Thus, when the noise occurs, the thin plate oscillates and propagates acoustic pressure while barely passing through the core panel. The effectiveness of the unit cell hexagonal cross section was evaluated to predict the acoustic characteristics of the AMSes. Through Rayleigh′s method of a spring and a mass, the effective dynamic mass density, *ρ_eff_*, can be determined using the following deceptively simple equation [19,20]:

(1) $$\rho_{eff}=\;\rho_{m}\left(1-\frac{f_{r}^{2}}{f^{2}}\right)$$

where *f_r_* is the lowest eigenfrequency of a honeycomb-shaped thin plate, *f* is the sound frequency, and *ρ_m_* is the density of the thin plate. The given equation originates from Newton′s second law, but the dynamic inertial mass of the system becomes a function of frequency due to internal mass and spring interactions [19]. Thus, the dynamic mass density differs from the conventional gravitational mass density. In the case of the hexagonal clamped thin plate, *f_r_* is calculated using the following equation [30,31,32]:

(2) $$f_{r}=\frac{\pi \alpha }{6a_{m}^{2}}\sqrt{\frac{D}{\rho h_{m}}},\;\;D=\frac{E_{m}h_{m}^{3}}{12\left(1-\nu_{m}^{2}\right)}$$

where *h_m_* is the thickness of the thin plate, and *a_m_* is the side length of the thin hexagonal plate. *E_m_* and *ν_m_* represent Young’s modulus and Poisson’s ratio of base material for the thin plate, respectively. The constant *α* is a nondimensional frequency parameter calculated by the energy approach and convergence study [32]. For the first mode, *α* is 3.9068. If *f < f_r_*, the frequency-dependent effective dynamic mass density becomes negative. This implies that the force and the acceleration have the opposite direction. The clamped thin plate′s local oscillation provides the anti-resonance, which is out of phase with the incident wave. Therefore, the acoustic wave through the thin plate ceases to propagate and becomes evanescent, as the negative density implies an imaginary wave vector.

**Figure 2:**
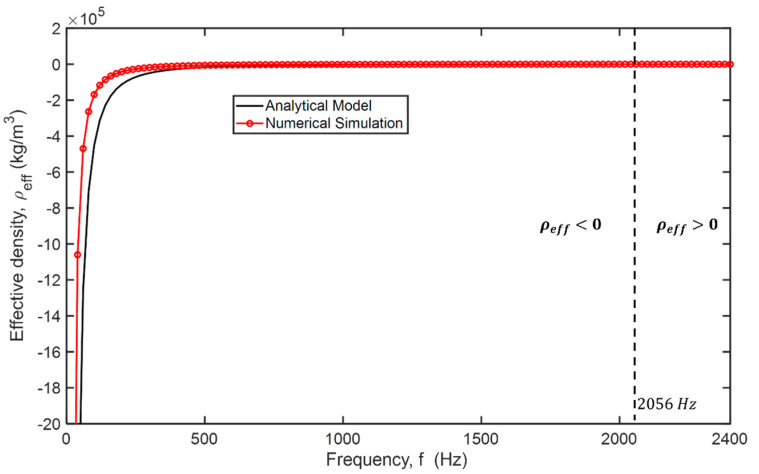
The clamped hexagon plate′s dynamic mass density is calculated by the analytical model (solid black line) and the numerical simulation (solid red line with marker). The fundamental resonance of the thin plate is at 2056 Hz.

The effective dynamic mass density of an AMS, including an elastomeric thin plate having an eigenfrequency of 2,056 Hz, was obtained from Equation (1) and the numerical simulation using COMSOL Multiphysics when *h_m_ = 0.5 mm*, *E_m_ = 7 MPa*, *ρ_m_* = *1070 kg/m^3^*, *ν_m_ = 0.49*, and *a_m_ = 3.65 mm* (see Figure 2). The effective dynamic density can be numerically obtained by dividing the out-of-plane surface averaged stress, $\bar{\sigma_{yy}}$, by the product of the surface averaged acceleration, $\bar{a_{y}}$, and the thin plate thickness, *h_m_*, i.e., $\rho_{eff}=\bar{\sigma_{yy}}/\left(\bar{a_{y}}h_{m}\right)$ where the y-axis is the direction of wave propagation [20].

### 2.2. Fabrication

**Figure 3:**
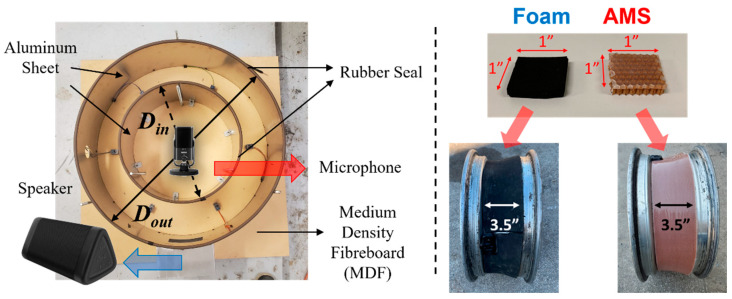
(**Left**) Tire cavity model representing a tire, 235/65R18. At the bottom, there is a hole for the speaker which generates white noise to represent TPIN. The Rode NT-USB mini microphone was mounted in the center of the inner cavity and connected to the computer with the USB cable. (**Right**) The rim of the tire (Pirelli Tire), 185/65R15, of Toyota Prius hybrid 2008. The foam and the AMS were bonded on the circumference of the rim, 3.5″ width.

To demonstrate the noise reduction of AMSes, we used the AMSes attached tire cavity model and conducted a field test with tires covered with AMSes to prove their noise reduction capability. Based on the parametric study (see Section 3.1.), we first fabricated AMSes made up of a honeycomb core panel and silicone rubber. The aramid honeycomb core panel has a 1/8″ cell with a density of 3 lb/ft^3^ and 1/4″ thickness (ACP Composites, Inc, Livermore, CA, USA). The silicone rubber, called the dragon skin (Smooth-On, Inc, Easton, PA, USA), is low viscosity cure silicone that does not require vacuum degassing. The silicone rubber consists of two resins which should be mixed at a ratio of 1 to 1. To place the thin rubber plate on the core panel, first, the silicone rubber was poured as evenly as possible on the clean surface of the wood plate like a mold, which was prepared with a 0.04″ (1 mm) deep channel utilizing a CNC router. It was then smoothed over with an 11″ paint shield which rested on the edges of the channel, resulting in a thickness of the silicone rubber of about 0.04″. Next, the quarter-inch-thick honeycomb core panel is put on the rubber layer and cured for 2 hours at room temperature, i.e., the silicone rubber covered one side of the panel (see Figure 3 (right)).

Various experimental techniques have been used to measure the soundproofing capability in the lab, e.g., directly measuring sound with microphones in the cavity resembling the actual structures [7,33], or indirectly measuring vibration of the structures induced by the impact [34]. The cavity model was used for the lab test due to its simplicity and convenience. The manufactured AMSes were attached to the inner layer of the tire cavity model, mimicking a real tire, 235/65R18, for the lab test referring to O’Boy’s design [7], and the rim of each tire (Pirelli Tires), 185/65R15, of the Toyota Prius hybrid 2008, for the field test. The tire cavity model consisted of medium density fibreboard (MDF), aluminum metal sheets, and acrylic panels, as shown in Figure 3 (left). The outer and the inner metal sheets represent the tire rubber and the rim of a wheel where the outer and inner diameters are *D_o_* = 30″ and *D_i_* = 18″, respectively. The thickness values of Al sheets, MDF, and acrylic panels, are 0.060″, 0.750″, and 0.437″, respectively. We added a rubber seal to the edge of the aluminum sheets to isolate the cavity. The Rode NT-USB mini microphone (RØDE Microphones LLC, Long Beach, CA, USA), which has a sampling rate of 48 kHz and a frequency range of 20 Hz–20 kHz, was mounted in the center of the inner cavity and connected to the computer with the USB cable. A hole was made at the bottom for a speaker emitting white noise which was generated by the Minirator MR2 audio generator from NTi Audio (NTi Audio AG, Schaan, Liechtenstein), which has a resolution of 0.1 Hz. The foam and the AMS were bonded on the circumference of the rim, 3.5″ width. The densities of the foam and the AMS are approximately 10.0 lb/ft^3^ and 14.5 lb/ft^3^, respectively.

For the field test, AMSes were bonded on the rim inside each tire of the vehicle (see Figure 3). A commercial soundproofing foam (neoprene sponge foam rubber from Lazy dog warehouse) with the same thickness of the honeycomb core panel was used as a comparison.

## 3. Results

**Figure 4:**
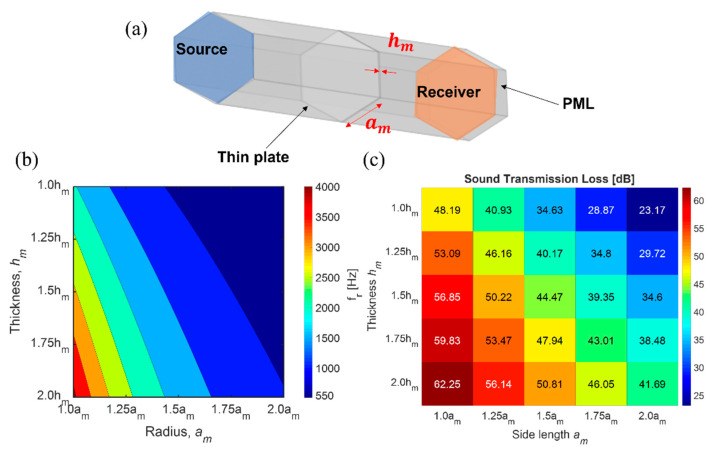
The result of the parametric study on design parameters, such as thickness, *h_m_*, and side length, *a_m_*, of the thin plate of the unit cell of AMSes; (**a**) the schematic image of the unit cell used in Figure 1 and Figure 2; (**b**) the natural frequency of AMSes; (**c**) the average STL of AMSes from 100 Hz to 400 Hz calculated by the numerical simulation.

A parametric study of the unit cell was conducted to evaluate the effects of design parameters, such as side length and thickness of the unit cell′s thin plate, density, and sound transmission loss (STL) (see Figure 4). Then, AMSes were fabricated based on the parametric study to maximize STL yet remain lightweight and were attached to the tire cavity model and an actual tire for the laboratory and field tests. The performance of the AMSes was compared to a commercial foam with the same thickness.

### 3.1. Design Map of the Unit Cell of the AMS

The unit cell′s parametric study was carried out using the numerical simulation (COMSOL Multiphysics) with the pressure acoustics and the solid mechanics modules to predict the effect of the design parameters on acoustic properties, such as dynamic mass density and STL. The thickness, *h_m_*, and side length, *a_m_*, of the thin plate were examined. The clamped hexagonal thin plate was considered the unit cell of the AMS, and the linear elastic model for the silicone rubber was occupied. Figure 4a illustrates the geometry of the unit cell, where the thin plate is placed in the middle of the pipe. The maximum element size of the pipe is 0.1λ where λ is the wavelength. The number of elements along the thickness of the thin plate is five. Before and after the thin plate there are boundary layers regarding viscous layer thickness. The perfectly matched layer (PML) is placed on the back of the receiver side to avoid the reflection from the wall. The boundary condition of the sidewall is hard wall. On top of the pipe, the plane wave propagates through the structure. Then, the transmitted sound pressure was measured at the bottom.

The peak of the noise is near 230 Hz, which is the fundamental mode of the tire cavity. The investigation’s frequency range was from 100 Hz to 400 Hz, to consider the effects on the fundamental mode. As mentioned above, when the frequency is less than the fundamental mode of AMSes, the effective density of AMSes becomes negative. Under these conditions, the plate’s local oscillation reflects the incident wave resulting in a substantial noise reduction. Therefore, when the natural frequency is shifted to a higher frequency by modifying the design parameters, the noise reduction effect is enhanced (see Figure 4b,c). Figure 4c shows the average STL of AMSes from 100 Hz to 400 Hz, depending on a variety of design parameters. The AMS shows a significant noise reduction of 23–62 dB at the low-frequency ranges, even though the material properties of the silicone rubber were simplified in this study using a linear elastic model. A smaller unit cell and a thicker plate have even higher sound losses because the first mode is proportional to the thickness and inversely proportional to the plate′s area (see Equation (2)).

### 3.2. The Sound Pressure Level in the Tire Cavity Model (Static Test)

**Figure 5:**
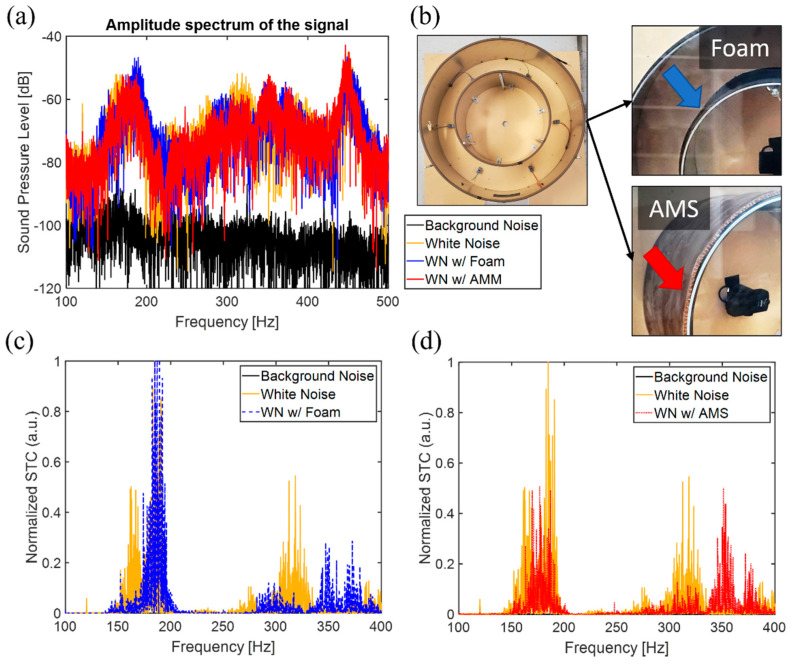
Sound pressure level (**a**) and normalized sound transmission coefficients (**c**,**d**) in the tire cavity model measured in the inner cavity. The background noise, black solid dashed lines, is the reference. The yellow lines represent the white noise (WN) when the speaker is turned on. The blue and red lines represent the attached foam and AMS, respectively. The pictures (**b**) display the cavity models with foam or AMS utilized in the experiment.

The AMS feasibility was demonstrated by constructing a tire cavity representing an actual tire (235/65R18), as shown in Figure 3. The model consisted of MDF, aluminum metal sheets, and acrylic panels. The outer and the inner metal sheets represent the tire rubber and the rim, respectively. The Rode NT-USB mini microphone was mounted in the inner cavity. A hole at the bottom facilitated the generation of white noise via a speaker. Figure 5 shows the effects of noise reduction due to the AMSes. Figure 5a shows the acoustic spectrum in log scale, and Figure 5c,d depict the sound transmission coefficients (STCs) normalized to the maximum sound transmission of the white noise of the cavity mode. Figure 5b illustrates the tire cavity with foam and AMS.

In Figure 5, the solid black line represents background noise, and it serves as a reference. The speaker generating white noise (WN) was turned on to measure the spectra of the empty inner cavity and is depicted by the yellow lines. Due to the circular symmetry of the tire cavity, there are radial and azimuthal eigenmodes. The dominant contribution to automobile noise originates from the fundamental radial mode with a frequency near 184.7 Hz. There is a peak near 185 Hz in Figure 5. The suppression of this peak noise in the frequency spectrum within the inner cavity strongly reduces the noise transmitted to the car′s cabin. The efficiency of our metasurface-based technology is compared with existing sound absorption-based noise reduction technology. The noise in a cavity filled with 1/4″ thick foam (shown by the blue dashed lines) is compared with the cavity wrapped using the acoustic metasurface (shown by the red dotted lines). It is evident from the acoustic spectra shown in Figure 5c,d that the noise within the inner cavity is reduced in both cases. The reduction is more substantial in the case of the AMS, especially near the cavity mode (about twice (3 dB) that of the foam), and it remains effective over a broader range of frequencies. The bandwidth of the noise suppression frequency is narrower for the foam as it continues to transmit sound energy while absorbing due to thermal dissipation. The wavelength at the low frequency is much larger than the porous size of the foam. The AMS, on the other hand, reflects due to anti-local resonance below the natural frequency of the thin plate. There are several minor peaks at higher frequencies of 350 Hz, and the unit cell design can suppress that. As the dynamic mass density is a function of design parameters, the modes can be varied or specified to reflect and consequently reduce noise.

### 3.3. The Sound Pressure Level in the Cabin (Dynamic Test)

**Figure 6:**
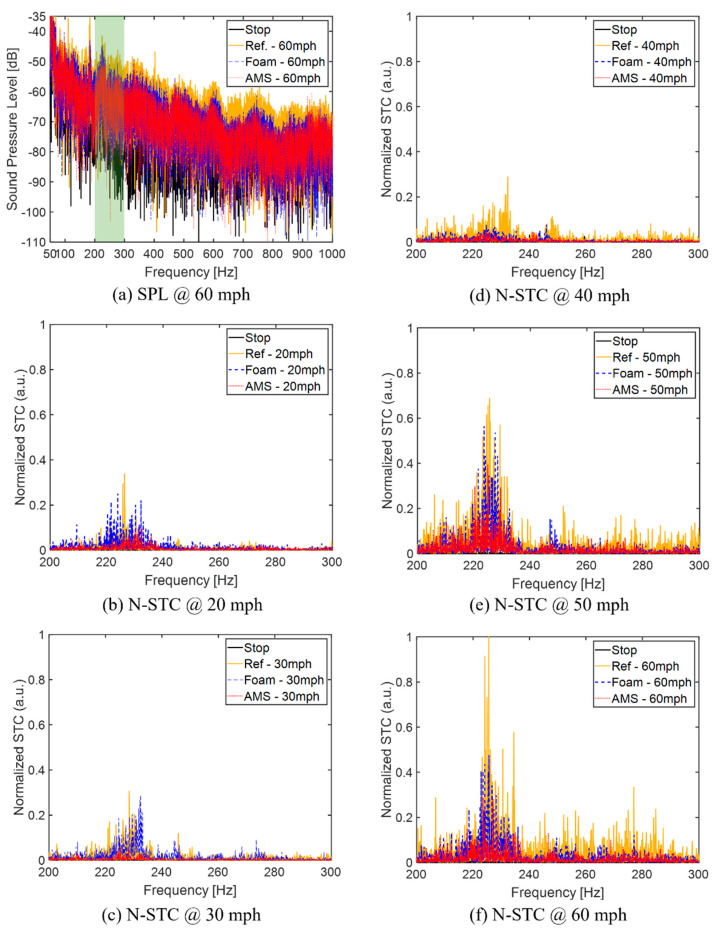
The result of the sound reduction performance of the AMS (red) and the foam (blue) compared to the original tire (yellow) without any attachment through the field test. (**a**) The sound pressure spectra from 100 Hz to 1000 Hz. The solid black line represents the stopped state. At 60 mph, yellow (solid), blue (dashed), and red (dotted) lines represent no attachment, foam attachment, and AMS on the rim, respectively. (**b**–**f**) The normalized sound transmission coefficients (N-STC) from 200 Hz to 300 Hz depending on the vehicle speed.

**Figure 7:**
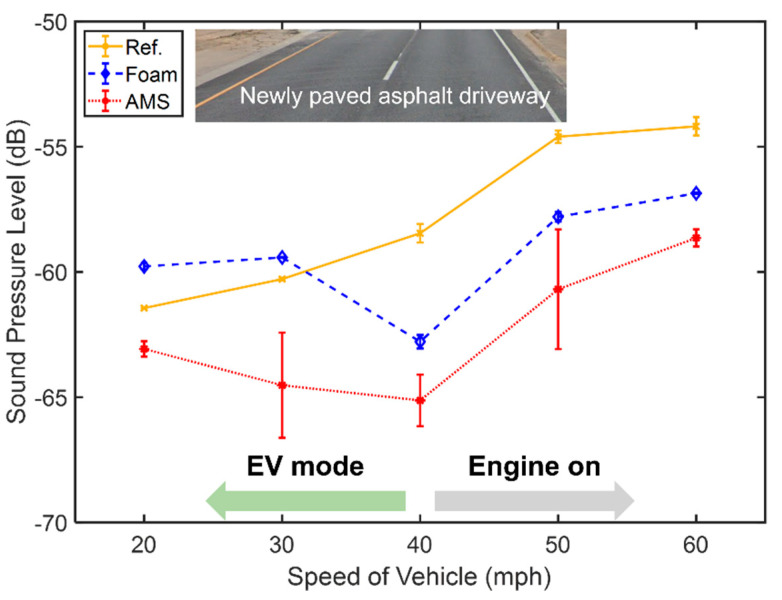
The average sound pressure level from 200 Hz to 300 Hz versus the vehicle’s speed with error bars. Yellow (solid), blue (dashed), and red (dotted) lines represent no attachment, foam attachment, and AMS on the rim, respectively. The inset depicts the newly paved asphalt driveway.

After the lab-scale test, the field test was performed with the Toyota Prius hybrid 2008. This car was chosen to minimize the noise induced by engine and exhaust and focus on the tire–pavement interaction. The Prius hybrid has an electric vehicle (E.V.) mode up to 60 km/h (~37 mph). The tire size is 185/65R15 (Pirelli Tire), and the air pressure in the tire is 44 psi. To investigate the effectiveness of AMSes, we consider three cases—(i) without any attachment, (ii) with foam, and (iii) with AMS (Figure 6). The test was performed on the local driveway, about 7.2 miles, newly paved with asphalt in 2020 (see inset to Figure 7). The noise inside the cabin was measured twice from 20 to 60 mph with a 10 mph interval.

The temporal sound pressure was measured for 19 s with a Rode NT-USB mini microphone, and the recorded data were processed with the fast Fourier transform (FFT) to obtain the frequency spectrum of the sound pressure level (SPL) from 50 Hz to 1,000 Hz as shown in Figure 6a. The cavity mode occurs near 230 Hz, as expected. Although both foam and AMS show noise reduction effects, the SPL of AMS is 2–3 dB more than foam and is significantly higher. The frequency range under consideration ranged from 200 to 300 Hz as the tire air cavity mode appears near 230 Hz. The frequency spectrum of the sound transmission coefficient (STC) at various vehicle speeds is shown in Figure 6b–f. STC is normalized by the peak of the cavity mode at 60 mph. For low speed at E.V. mode, 20–40 mph, the cavity mode′s peak values are similar but the noise at other frequencies induced by engine increases as the vehicle speed increases. 

The average sound pressure level in the band 200–300 Hz depends on the vehicle speed, as shown in Figure 7. The noise level increases with the speed, and the slope of noise changes when the mechanical engine kicks on after 40 mph due to the electric to gasoline power modes. Average values are used so that the foam case shows more noise than the reference case. Although the foam reduces the cavity mode’s noise, more peaks occur near the cavity mode, as seen in Figure 6b. The AMS is 1.45 times heavier than the foam. Nevertheless, through all speed ranges, the AMS clearly provides a better noise reduction effect than the foam, 2–5 dB near the cavity mode, at 200–300 Hz.

## 4. Further Discussion

The acoustic foam is generally used to reduce noise, not only for tires but also for stationary structures, e.g., in civil engineering as an absorber [35,36,37,38]. For the tire application, Mohamed et al. validated the effectiveness of foam [39]. However, the absorption coefficient is less than 7%, and even lower at the low-frequency range. As a reflector, the AMS displays a better noise reduction capability, about 2–3 times (~3 dB) that of foam at the low-frequency range, in the region of the cavity mode, and the broadband. However, the actual effectiveness of the application (~10 dB) is much lower than the preliminary results (23–62 dB) from the unit cell; the AMS can only cover 3.5″ of the rim due to the non-flat surface, so noise induced by TPIN continues to propagate through the uncovered area. With a modified and optimized design concerning the rim, an adequate bonding mechanism between the rim and the AMS for durability, and a suitable material selection, the efficiency of AMS noise reduction could be improved substantially.

## 5. Conclusions

We propose a noise-reduction method for tires using relatively lightweight acoustic metasurfaces or metalayers with high reflective and absorbing characteristics. The natural frequency of the clamped thin plate is calculated by the numerical simulation and validated by the references. The effectiveness of the AMS was demonstrated numerically and experimentally, but these were not compared to each other directly. The design guideline was provided through the parametric study. Our in-house AMS prototype was utilized for the experiment both on the laboratory scale and for actual field tests in a hybrid car. The noise reduction effect of the AMS can be manipulated by tuning the thin plate’s fundamental resonance via certain design parameters. The method in this work can substantially reduce the noise near the tire cavity mode at around 230 Hz and extends over a broader range of frequencies under 1000 Hz. The field test results demonstrated that almost-total AMS reflection provides a better noise reduction effect than absorption by foam, 2–3 dB near the cavity mode at 200–300 Hz. Even considering that AMS is slightly heavier than foam, the former is still 1.4–2 times more effective than the latter when it comes to noise reduction.

Furthermore, the structure of the material used in this approach is lightweight and does not affect the tire′s performance. It can be easily combined with the existing technologies to maximize the sound transmission loss. The robust and lightweight nature of the design means that it can be modified and applied to other fields for sound and vibration isolation to reduce noise issues.

## Data Availability

Data is contained within the article.
